# Exploiting Benefits of Vaterite Metastability to Design Degradable Systems for Biomedical Applications

**DOI:** 10.3390/pharmaceutics15112574

**Published:** 2023-11-02

**Authors:** Yulia Svenskaya, Tatiana Pallaeva

**Affiliations:** 1 Scientific Medical Center, Saratov State University, 410012 Saratov, Russia; 2 FSRC “Crystallography and Photonics” RAS, 119333 Moscow, Russia

**Keywords:** calcium carbonate, CaCO_3_ particles, vaterite, metastability, recrystallization, calcite, degradation, dissolution, pH-sensitivity, resorption, biodegradation, controlled release, ayer-by-layer assembly, calcium ions, carbon dioxide bubbles, ossification, theranostics, anticancer therapy, antimicrobial therapy, US imaging, cavitation, buffering

## Abstract

The widespread application of calcium carbonate is determined by its high availability in nature and simplicity of synthesis in laboratory conditions. Moreover, calcium carbonate possesses highly attractive physicochemical properties that make it suitable for a wide range of biomedical applications. This review provides a conclusive analysis of the results on using the tunable vaterite metastability in the development of biodegradable drug delivery systems and therapeutic vehicles with a controlled and sustained release of the incorporated cargo. This manuscript highlights the nuances of vaterite recrystallization to non-porous calcite, dissolution at acidic pH, biodegradation at in vivo conditions and control over these processes. This review outlines the main benefits of vaterite instability for the controlled liberation of the encapsulated molecules for the development of biodegradable natural and synthetic polymeric materials for biomedical purposes.

## 1. Introduction

Over the recent decades, calcium carbonate-based materials have gained a tremendous interest in a broad range of biomedical applications [1]. Being degradable, biologically compatible and low-cost, CaCO_3_ is widely used to manufacture drug delivery systems [2,3,4], biosensors [5], tissue-engineering scaffolds [6,7] and imaging platforms [8,9]. The low toxicity and unique physico-chemical properties of this inert material make it suitable for various routes of administration, whether gastrointestinal, parenteral or topical [2]. Thus, for instance, previous studies have considered its intravenous [10], intramuscular [11] and subcutaneous [12] injection, as well as oral [13], nasal [14], dermal (e.g., intradermal [15] and intrafollicular [16]) and even tracheal [17] administration for the delivery of CaCO_3_ particles. Calcium carbonate is a highly available material since it abundantly occurs in nature as a component of limestone, marble and chalk in sedimentary rocks and as a content of marine sediments [18] and spring deposits [19]. Furthermore, it can be easily synthesized in the lab choosing the most suitable protocol among a wide range of methods [18,20]. CaCO_3_ appears in living organisms, e.g., as a component of bones, teeth, shells, coral skeletons and eggshells [21,22] and can also be produced by various bacteria [23], which has opened up the great opportunities for biomimetic synthesis of this material to make it even more safe and compatible for biomedical applications.

Calcium carbonate presents the phenomenon of polymorphism and appears either in crystalline solid forms of anhydrous (calcite, vaterite and aragonite) and hydrated (ikaite and CaCO_3_ monohydrate) polymorphs, or in amorphous calcium carbonate (**ACC**) modification [24]. Cubic calcite crystals with rhombohedral lattice and needle-like aragonite crystals with orthorhombic lattice are more thermodynamically stable and, thus, represent the most widespread form of anhydrous CaCO_3_ [25]. In contrast, mesoporous vaterite polycrystals and ACC are non-stable and can only be found in nature when their surface is stabilized with some additives [26]. In spite of, and even benefitting from such instability, these two forms are highly demanded in biomedicine. ACC comprises the seeds for crystal growth of the other CaCO_3_ polymorphs and plays a significant role in biomineralization processes [27]. Thus, ACC clusters are effectively used to design the implant materials and coating for the implants [28,29]. Vaterite is metastable and the most soluble CaCO_3_ polymorph [30]. This material is widely applied to create novel vehicles for drug delivery and templates for therapeutic platforms with a broad range of biomedical applications [2,4].

The crystal structure of vaterite is long being debated. Kabalah-Amitai et al. showed that this form of CaCO_3_ contains at least two coexisting crystallographic structures forming a pseudo-single crystal [31]. In particular, they stated that vaterite represented a hexagonal lattice structure with the nanodomains of an unknown structure distributed within its matrix. Vaterite rarely occurs as single crystals (both in geologic/biominerals and when synthetically produced) and is often formed as spherulitic polycrystalline aggregates [32]. Due to this feature, vaterite particles are mostly obtained as mesoporous with a large surface area, which is usually around 20 m^2^g^−1^ [33,34], but can be increased even up to 200 m^2^g^−1^ by varying the reaction medium for its synthesis [35]. The porosity of this material allows for the incorporation of various substances making it especially advantageous in terms of drug, proteins and gene delivery [36]. It should also be noted that vaterite particles are used as carriers on their own or as a part of a composite (hydrogels, fibers and implanted materials), where it is incorporated in order to improve mechanical or therapeutic functions.

Owing to its instability, vaterite can be either transformed into non-porous calcite crystals via dissolution–reprecipitation [37] or even completely dissolved/resorbed, depending on the immersion medium used [16,38]. The release of the incorporated cargo is driven by such transitions. Importantly, the rate of these processes strongly depends on the surrounding conditions, such as the pH [39], temperature [37] and ionic strength [40] of the media and presence of different ions or additives [26,41,42], thus can be controlled externally. Furthermore, depending on the intended use of the vaterite carriers, the payload liberation can either by delayed by means of their surface modification [43,44] or accelerated, e.g., by means of ultrasound treatment [45]. In addition to the transition-driven drug release property, vaterite particles can serve as a source of Ca^2+^ ions. This feature is effectively exploited when creating the scaffolds for bone and tooth tissue regeneration [6,46] due to the ability of calcium ions to improve osteo- and odontoblasts’ activity [47]. Moreover, such capability of vaterite to release Ca^2+^ ions is of high importance when designing hydrogels with an autogelation property as far as these cations can efficiently bind polymer chains in hydrogels providing the hydrogel formation [48,49]. In addition, this feature is extensively used to create CaCO_3_-based hemostatic materials [18,50,51].

Being degradable at mild conditions, vaterite particles are also used as sacrificial templates for the fabrication of other functional materials and biosensors [52]. For instance, layer-by-layer adsorption of biocompatible polyelectrolytes onto these particles together with further dissolution of vaterite cores allows one to fabricate bio-friendly hollow polymer capsules [53]. Vaterite-based templating is also utilized to design porous alginate hydrogels with a well-controlled architecture aiming at fabrication either of drug delivery systems or three-dimensional cell scaffolds [49]. Dissolution of the template in such systems can be achieved by complexation with ethylenediaminetetraacetic acid (**EDTA**) at a neutral pH [54] or by reducing the pH [55] due to the feature of vaterite to decompose rapidly under acidic conditions [38].

Another important outcome of vaterite instability is associated with the generation of carbon dioxide bubbles during dissolution in acidic media. This property determines the potential of CaCO_3_-based carriers’ application in ultrasound imaging and therapy [18]. Besides, the dissolution of vaterite in an acidic environment can increase the local pH due to its regenerative buffering capacity [56]. This effect was shown promising for the use in anticancer therapy, as it enables modulation of the extracellular pH in tumors inducing the cellular metabolic reprogramming [57].

The numerous advantages that we listed above explain the high interest of researchers in calcium carbonate-based materials. To date, a great number of comprehensive reviews have been published that summarize the synthesis techniques and protocols, discuss the main applications of CaCO_3_ and offer different perspectives on this object [2,3,18,42,58,59,60,61]. Nevertheless, none of them emphasized vaterite separately and suggested that attention should be paid to the possibility of using the metastability of this material to advantage. In view of this fact, our review highlights the main benefits of vaterite instability potentiating its employment in the design of degradable systems for biomedical purposes.

## 2. Incorporation of Various Substances into the Vaterite Matrix

**Figure 1:**
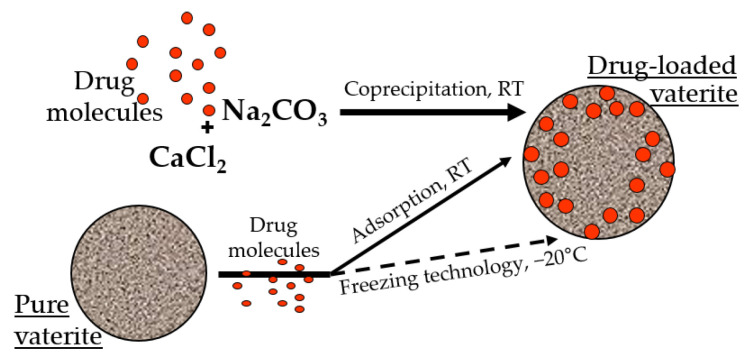
Incorporation of the drug molecules within the vaterite particles by adsorption and co-precipitation methods at room temperature (RT), and via freezing induced loading at −20 °C.

There are two main approaches for the entrapment of functional substances into the calcium carbonate particles, namely: sorption [53] and co-precipitation [62] (Figure 1). The first one is based on the inclusion of drug molecules as a result of their physical sorption in the pores of preformed CaCO_3_ matrices. In the co-precipitation method, the formation of carbonates occurs in the presence of an active compound resulting in vaterite crystallization with simultaneous inclusion of the active molecules. Both techniques allow the co-immobilization of several bioactive compounds within one particle [2].

It was demonstrated previously that the loading efficiency by the co-precipitation is higher than by the sorption, especially for high molecular weight molecules of a hydrophilic nature [63,64]. It is probable that during the formation of the particles in the presence of the drug molecules, the encapsulated substance is distributed throughout the entire volume of the carbonate matrix, and during physical sorption, it occurs mainly on its surface [65]. Taking into account the stability of the calcium carbonate in non-polar solvents, the surface sorption of the biologically active substances allows the loading of hydrophobic compounds, which is restricted for the co-precipitation approach [66,67,68]. Moreover, the entrapment efficacy of the vaterite particles could be enhanced by several methods, including a freezing technology, where successive cycles of freezing and thawing resulted in the substance embedment in the particles’ pores by the growing pressure of the forming solvent crystals [69,70,71,72]. Various additives during particle synthesis were also utilized to intensify the loading capacity of the carbonate matrices, such as proteins [73,74,75,76], polysaccharides [77,78,79,80,81], glycosaminoglycans [82], glycoproteins [63,83], etc. [84,85,86].

To date, almost all known classes of substances have been successfully loaded into calcium carbonate particles, including but not limited to herbal extracts [65,87], genetic materials [88,89], vaccines [90,91], enzymes and other proteins and peptides [92,93,94,95,96], anticancer drugs [58,97], including photosensitizers [38,98] and therapeutic radionuclides [99], antimicrobial compounds [43,100,101,102] and others [103,104].

## 3. Vaterite Recrystallization to Calcite: Mechanism and Associated Release of the Loaded Drugs and Calcium Ions

**Figure 2:**
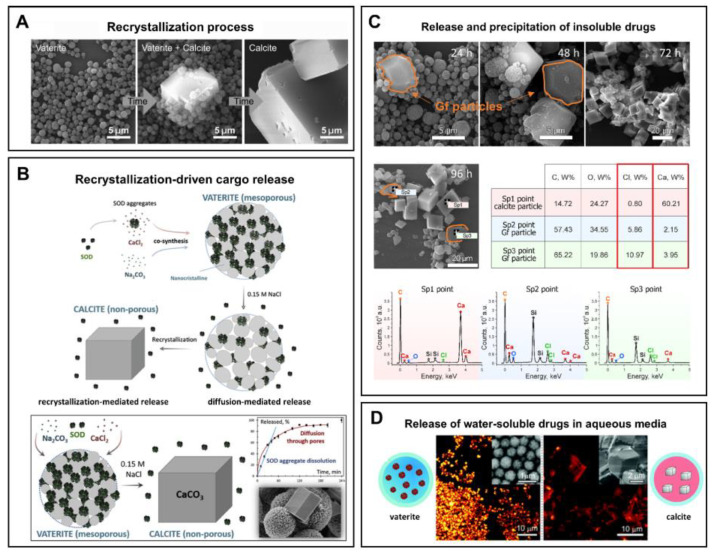
(**A**) Schematic representation of the vaterite–calcite recrystallization process. Reproduced with permission from [101]. (**B**) Schematics for the process of drug liberation from vaterite carriers, which is mediated by the vaterite–calcite recrystallization. Reproduced from Open Access Article [107]. (**C**) Release of water-insoluble drugs from vaterite carriers in aqueous media resulting in the formation of insoluble crystals (particles) by payload molecules. SEM images and results of EDX analysis illustrating the degradation process of the carriers loaded with griseofulvin (Gf) antifungal drug in deionized water. The precipitated Gf particles are contoured with orange. Reproduced with permission from [43]. (**D**) Release of water-soluble drugs from vaterite carriers in aqueous media. Schematics of the release process and corresponding two-photon fluorescence microscopy and SEM (insets) images of the carriers loaded with rhodamine 6G before and after their incubation in water. Adapted with permission from [33].

The instability of vaterite manifests itself in contact with water. Being quite stable in the dry state, it dissolves/recrystallizes upon incubation in aqueous solutions [30,105]. In particular, under non-acidic conditions, vaterite easily and irreversibly transforms into calcite form [106]. This transformation takes place through the dissolution of vaterite followed by the nucleation and growth of the calcite crystals (solution-mediated transformation). Such a recrystallization process is gradual and starts at the surface of vaterite particles. Specifically, the external layer of the particles starts to solvate and ionize, the constituent ions (Ca^2+^ and CO_3_^2−^) diffuse away from the surface and then seeds the formation of calcite monocrystals [105]. In such a manner, porous spherical particles reassemble into smooth cubic ones, which are generally larger in size (Figure 2A).

The rate of the recrystallization process depends on the temperature and ionic strength of the immersion medium [37], as well as on the supersaturation level [108]. Namely, the vaterite–calcite transformation speeds up when these parameters increase. Specifically, at a higher temperature and ionic strength, the ion exchange between the particle surface and the incubation solution is accelerated, which leads to the faster transition to calcite. Relatively high supersaturation ratios (1.5–1.9) also speeds up this transformation as it is controlled by the vaterite dissolution in this case, whereas at lower supersaturation ratios (1.2–1.5), the rate of dissolution of vaterite is similar with that of the crystallization of calcite [108].

The major practical benefits of the transformation process appears when the vaterite carriers are applied for drug encapsulation and delivery as it opens up the possibility of a degradation-driven release of the payload. It is well-demonstrated that liberation of the loaded molecules from the porous CaCO_3_ particles results from drug desorption and carrier recrystallization [33,38,107] (Figure 2B). Thus, the release profile represents an interplay of these two processes and strongly depends on the immersion medium [109]. In particular, when the solvent is not payload-specific, the desorption process is obviously slow. In contrast, intensification of this process occurs if a suitable solvent penetrates into the vaterite matrix dissolving the drug, which then diffuses faster in the medium [109]. In addition, vaterite carriers liberate the loaded molecules during their degradation while forming calcite crystals. The released drug can either diffuse into the solvent [43] (Figure 2D) or precipitate out (if its solubility is limited in this media) [43,110] (Figure 2C).

In such a manner, the recrystallization-driven release mechanism allows for control of the payload delivery time by changing the properties of the environment [109]. However, the release rate also depends on the molecular properties of the cargo (e.g., its molecular weight and ζ-potential) [33], carriers’ size [38], and method of its loading into vaterite carriers (as this determines the filling density of the particles) [95]. Obviously, the lower the molecular weight of the payload, the smaller the size of the vaterite carriers and the more superficial the drug distribution across the carrier, the faster release occurs.

**Figure 3:**
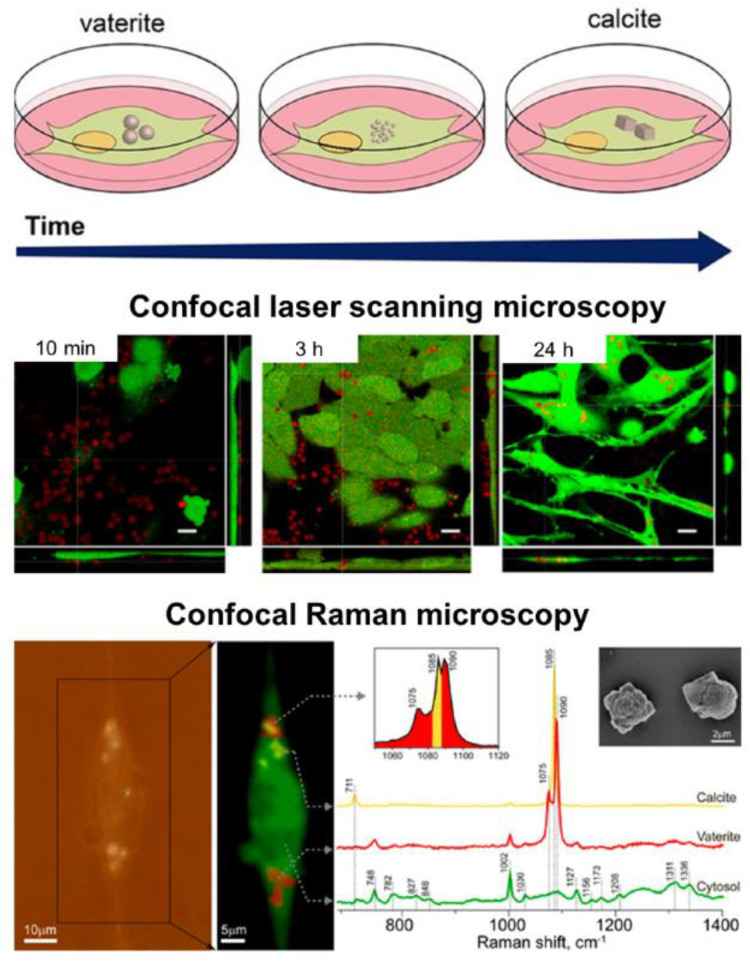
Intracellular recrystallization of vaterite carriers. Schematics of the vaterite–calcite transformation (**the upper row**), CLSM images of HeLa cells after their incubation with the carriers for 10 min, 3 and 24 h (**the middle row**) and the results of Raman analysis of a single cell after 72 h incubation with the carriers (**the bottom row**). Adapted with permission from [111].

The virtue of vaterite–calcite recrystallization is successfully employed for intracellular drug delivery. Thus, for instance, Parakhonskiy et al. have demonstrated the possibility of delivering drugs into living cells by means of vaterite carriers exploiting the delayed burst-release mechanism [33]. Furthering this line of research, this team has studied the intercellular behavior of vaterite particles in the cellular cytoplasm [111] (Figure 3). In particular, they have monitored the process of vaterite recrystallization within the cell in real-time by means of confocal Raman and laser scanning microscopies. The formation of the stable calcite phase from the clusters of vaterite particles was registered after 72 h of their incubation with cells, confirming an ion-exchange mechanism of vaterite–calcite transformation inside the cell. Importantly, multiple cytotoxicity studies have revealed that vaterite particles demonstrated no significant influence on the viability or metabolic activity of different cell lines [33,112,113,114]. That defines the possibility of their application in cellular drug delivery [115,116,117].

Regarding the intracellular delivery, it is important to note that the immersion media might stabilize the particle surface affecting the crystal phase transition [114]. In particular, it was repeatedly demonstrated that the incubation of vaterite particles in cell culture medium leads to the adsorption of protein molecules from the medium onto their surface [118]. The protein corona formed on the surface of vaterite carriers as a result of such adsorption decelerates the process of their transformation into calcite and hence slows down the rate of the payload release [43,114,119]. This effect commonly occurs in biological fluids when the foreign materials are introduced into the body [120,121]. The beneficial impact of protein corona formation is especially evident in targeted drug delivery. For example, it has been shown that such a prevention of rapid release positively contributed to the drug localization within the cell upon uptake of vaterite carriers [114]. In terms of photodynamic therapy (**PDT**), such an effect enabled the controlled consequential cell destroying by the laser in a point-wise manner [116].

The payload can also affect the process of vaterite–calcite transformation. Thus, the incorporation of proteins into the vaterite matrix might stabilize it, slowing down the transformation to calcite [122]. Namely, the delivery of an antiproliferative lectin (the *Dioclea violacea* lectin, **DVL**) into cancer cells utilizing the recrystallization-driven mechanism resulted in a more pronounced therapeutic effect due to such stabilization, which provided a more constant release over time. The local increase in lectin concentration and a constant exposure of the cells to the lectin was supposed to be responsible for the superior effect observed upon the usage of DVL-loaded vaterite carriers in comparison with DVL solution.

**Figure 4:**
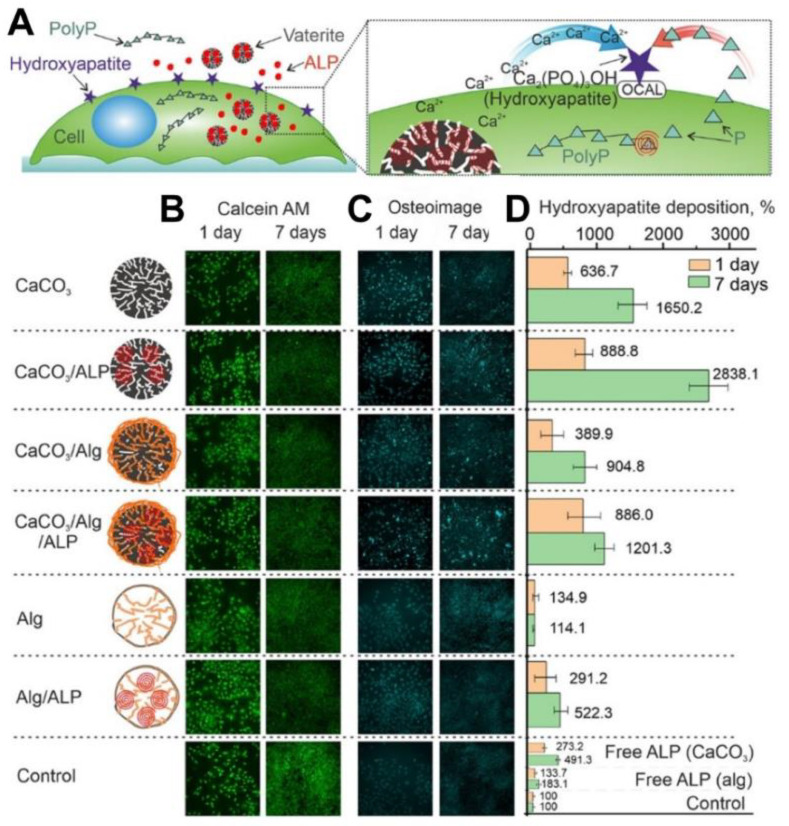
Exploiting of Ca^2+^ release, which occurs during the vaterite–calcite recrystallization, for improvement of the ossification process in vitro. (**A**) Schematic representation of the cellular treatment using vaterite carriers loaded with alkaline phosphatase (ALP). (**B**) Live cells stained by calcein AM (green), (**C**) fixed cells stained by the Osteoimage mineralization assay (Cyan) and (**D**) hydroxyapatite deposition measured at different times using the Osteoimage mineralization assay on MC3T3-E1 cells. Reproduced with permission from [123].

It is worth noting that in addition to the transition-driven drug release property, vaterite particles can serve as a source of Ca^2+^ ions while transforming to calcite. This feature has been recently exploited to accelerate the ossification both in vitro [123] and in vivo [6]. In particular, the immobilization and intracellular delivery of alkaline phosphatase (**ALP**) by means of vaterite carriers resulted in improvement of the ossification process in osteoblastic cells as the released ALP and Ca^2+^ ions represent essential components for extracellular matrix formation (Figure 4) [123]. The osteoinductive effect was demonstrated also in vivo when vaterite-coated polycaprolactone (**PCL**) scaffolds were loaded with ALP and implanted into a femoral defect in rats [6]. A significant increase in the osteoblast’s synthetic activity and intensification of bone tissue formation was observed due to the effective release of the enzyme and Ca^2+^ ions. This resulted in a complete restoration of the external defect cleft in the rat’s femoral bone.

**Figure 5:**
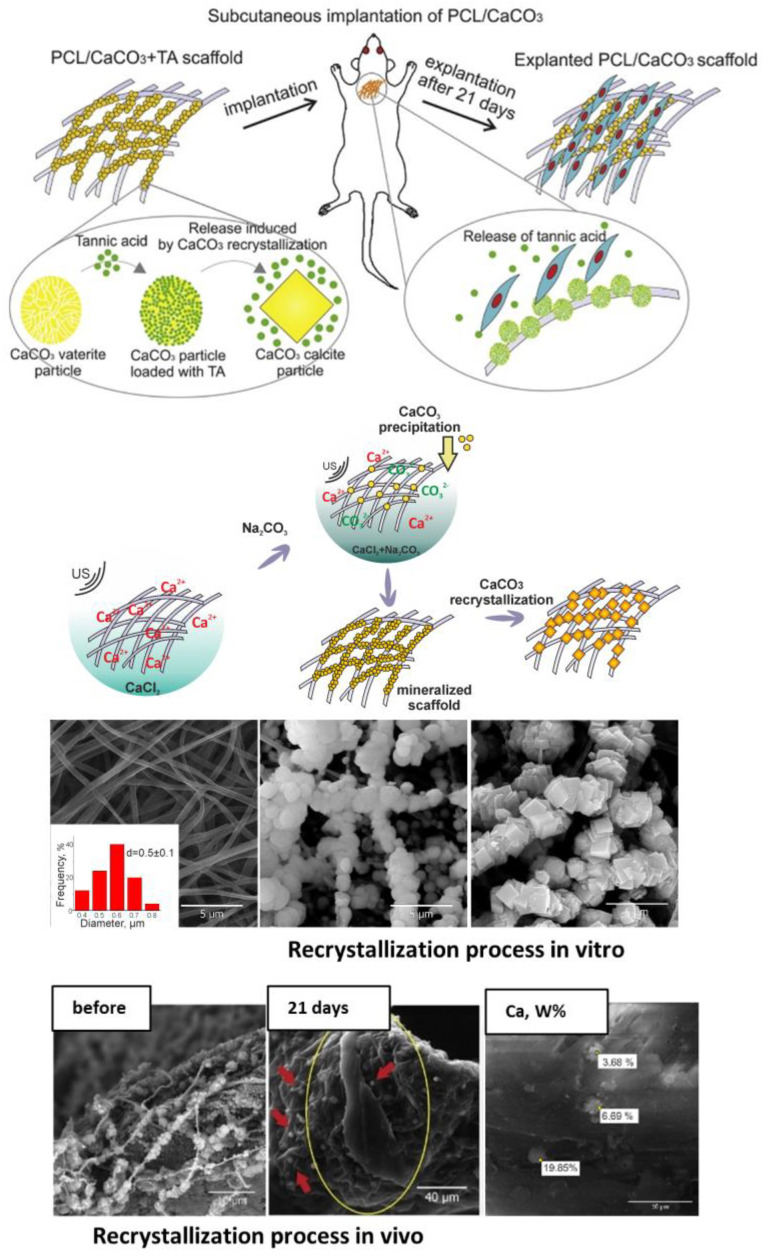
Recrystallization-driven drug release from the vaterite carriers in vivo. Schematic representation of tannic acid (TA) release from the vaterite-coated polycaprolactone (PCL) fibers subcutaneously implanted in rats (**the upper row**). Schematics and SEM images illustrating the process of the vaterite-coating formation and its transformation to calcite in an aqueous medium in vitro (**the middle row**). SEM images and results of EDX analysis illustrating the vaterite–calcite transformation of the fiber coating in vivo (**the lower row**). Reproduced with permission from [7].

Although vaterite recrystallization to calcite is generally observed in in vitro systems, the calcite formation can also occur during the degradation process at in vivo conditions [7,16]. In particular, at a high local concentration and dense arrangement vaterite particles can aggregate and recrystallize forming cubic-like crystals as the outflow of Ca^2+^ and CO_3_^2−^ ions from the carrier surface is not fast enough in this case. Thus, the above-described recrystallization-driven release property might remain actual for vaterite carriers when delivering drugs in vivo as well. For instance, Saveleva et al. demonstrated the liberation of tannic acid from the vaterite-coated PCL fibers in vivo, which took place through the vaterite–calcite recrystallization, lasted for 21 days when the scaffolds were subcutaneously implanted in rats (Figure 5) [7]. In our previous work, dealing with drug administration through the skin appendages (in particular, via hair follicles), in vivo monitoring reveals the active dissolution/recrystallization of vaterite carriers, resulting in their total resorption within 12 days [16]. The proposed particulate system served as an intrafollicular depot for a model drug storage and prolonged in situ release over this period.

It should also be mentioned that even though the main applications of the vaterite–calcite transition are related to the release of the loaded substance, such a transition can still be used, conversely, to incorporate different substances into CaCO_3_ particles [124,125]. In this case, the drug molecules are captured by calcite crystals formed during the incubation of vaterite particles in aqueous solution. This procedure can be applied when it is necessary to obtain the drug-containing calcite particles.

## 4. Vaterite Dissolution at Acidic pH: Mechanism, pH-Dependent Release of the Loaded Drugs, Calcium Ions and Carbon Dioxide Bubbles

**Figure 6:**
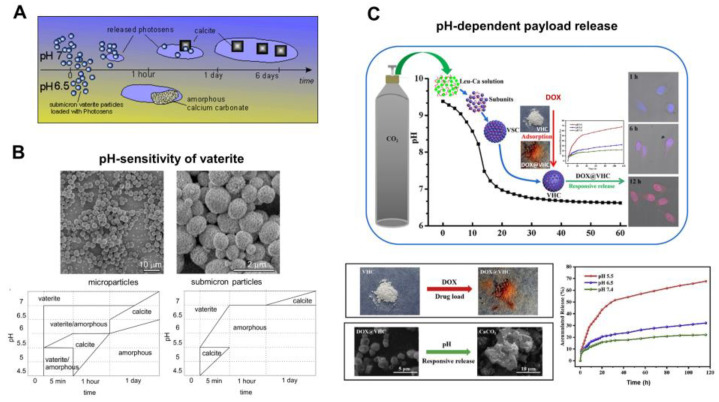
pH-dependent dissolution of vaterite carriers triggering the payload release. (**A**) Schematic representation of the decomposition-mediated drug release process from the vaterite carriers depending on the pH of the medium. Reproduced with permission from [38]. (**B**) SEM images of micro- and submicron vaterite carriers loaded with a photosensitizer before their incubation in various media and the phase-schemes illustrating the process of their transformation in acetate buffers (pH 4.5–6.5) and in water (pH 7.0) during 24 h. Reproduced with permission from [38]. (**C**) Schematics and SEM images illustrating the dissolution of vaterite-based carriers (VHC) and kinetics of the pH-dependent release of the loaded doxorubicin (DOX) drug. Reproduced with permission from [97].

Besides being transferable to calcite in neutral solutions, vaterite can also decompose rapidly with a decreasing pH (Figure 6A) [126]. For a quarter of a century, this property has been actively used to form hollow polyelectrolyte capsules [127,128]. Vaterite-templated consecutive adsorption of polyelectrolytes followed by the core decomposition is applied for the formation of polyelectrolyte micro- and nanocapsules of various shapes [53,129,130,131] aiming to deliver a great variety of payloads, from the fluorescent molecules to proteins [64], enzymes [132], different drugs [133] and genetic material [134].

Apart from that, vaterite particles themselves are used as the carriers releasing the loaded substances upon their decomposition in acidic media [44,61]. The dissolution of the carriers starts at their surface, where disintegration of the crystal lattice and hydration of constituent ions takes place [37]. Then, the dissolved matter is transported away from the particle surface into the bulk solution and the cargo molecules are liberated simultaneously. The dissolution-mediated release property granted the successful application of vaterite carriers in drug delivery and sensing [61]. Importantly, the dynamics of such release was demonstrated to be sensitive to the environmental pH [38].

In particular, in the work [38], the drug liberation process was studied by incubating photosensitizer-loaded vaterite carriers of two different sizes at room temperature in acetate buffers with a pH ranging from 4.5 to 6.5 and in deionized water with pH 7 for 6 days. It was found that vaterite particles dissolved rapidly with acidity increasing, as the CaCO_3_ solubility increases with pH lowering [135], and the amorphous phase appeared either before the recrystallization to calcite or as a final state (the phase-scheme is shown in Figure 6B). The time to complete vanishing of vaterite carriers decreased strongly with reducing the pH, so the photosensitizer liberation was increasingly dependent on the carrier dissolution process. A decrease of the particle size influenced the duration of their degradation in acidic buffers, where the complete dissolution of microparticles (3.6 ± 0.5 μm) was accomplished within 24 h of incubation at pH 6.5, while submicron particles (0.65 ± 0.03 μm) were completely dissolved within 1 h at the same pH. The fastest vaterite dissolution was observed at a low pH of 5 to 4.5, where the carriers of both sizes decomposed within the first 5 min causing an immediate burst release of the loaded photosensitizer. Meanwhile, at a neutral pH = 7, the photosensitizer release from vaterite submicron carriers lasted for several days and occurred during the transition to calcite.

Such a pH-dependent release of a payload from vaterite-based carriers was demonstrated multiple times by other authors. Thus, Feng et al. have shown that pH lowering leads to the greater amount of the liberated drug doxorubicin (**DOX**) from vaterite microparticles (~1.4 μm) [97]. Specifically, at pH 7.4 only 22% of the loaded DOX was released within 168 h, while at pH 6.5 liberation of 32% of the drug amount occurred for this period. When the pH level was set at 5.5, more than 40% of the payload released during 24 h and 68% liberated within 168 h. The authors proved that DOX release was induced by the carriers’ decomposition in an acidic environment, since transformation of the hollow vaterite structure to an amorphous form was observed (Figure 6C).

Yang et al. have demonstrated the possibility to trigger the release of the sanguinarine (**SAN**) anticancer drug from the vaterite carriers [136]. Comparison of the release behavior at pH 7 and pH 4 clearly demonstrated more sustained kinetics at the neutral conditions, where ~15% of the loaded drug appeared within 3.5 h and was followed by a slow release of 36% in the next 147 h. Meanwhile, at pH 4.0, these carriers exhibited a fast release of 72% in the first 3.5 h and the sustained release of up to 99% of the loaded SAN amount in the following 147 h. Moreover, vaterite exhibited a better pH-responsiveness than calcite illustrating the lower stability of the vaterite versus calcite crystalline phase. As mentioned above, this feature is an important advantage of vaterite which accounts for its wide application in biomedicine.

The same features were demonstrated for the hydrophobic drugs fluorouracil (**5-Fu**) and sodium levothyroxine (**L-Thy**) encapsulated into cyclodextrin (**CD**)-containing vaterite carriers [137]. Release studies demonstrated a more intense payload liberation upon the carrier incubation in acidic media (at pH 4.8 for 5-Fu and pH 1.2 for L-Thy) in comparison with a neutral solution (pH 7.4). The authors did not observe complete dissolution of the containers at such a low pH. This was most likely due to the stabilizing effect of the introduced CD. The pH buffering properties of CaCO_3_ could also be the reason for such observations. The dissolution of vaterite might increase the pH of the immersion medium affecting the further degradation of the carriers [56]. This feature should always be kept in mind when setting the mass of vaterite powder incubated and the volume of the immersion medium used, so that this effect can be leveled out.

Chesneau et al. have also demonstrated the pH-dependent release of the hydrophobic drug from CD-containing vaterite particles [67]. However, their carriers liberated the whole amount of the loaded tocopherol acetate (vitamin E) within 2 h while decomposing in acidic media at pH 5. This illustrates the importance of considering the leaching feature of vaterite. At pH 7.4 (0.15 M NaCl), the carriers remained stable and no hydrophobic cargo release was observed for this period.

**Figure 7:**
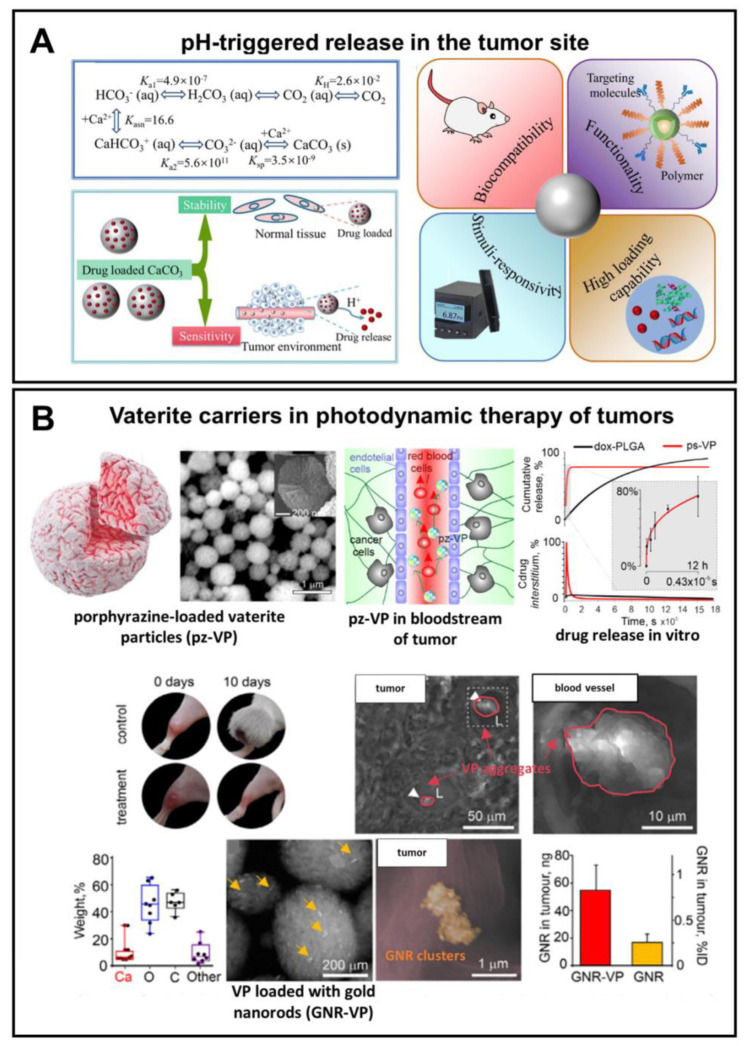
Exploiting pH-sensitivity of vaterite carriers for drug delivery to tumors. (**A**) Schematic presentation of vaterite application in tumor targeting. Reproduced from Open Access Article [18]. (**B**) An example of successful application of the vaterite particles loaded with a porphyrazine (pz) drug and gold nanorods (GNR) in photodynamic therapy of tumors. Adapted with permission from [98].

In terms of biomedical application, the pH-sensitivity of vaterite is of high importance in the targeted delivery of anticancer agents, since the microenvironment in tumors is generally more acidic than in normal tissues and in blood (pH 6.5–6.8 versus 7.4, respectively) [58,138] (Figure 7A). Similar to the other pH-responsive inorganic materials, vaterite can provide the pH-triggered release in the tumor site [139].

For example, by virtue of their pH sensitivity, Parakhonskiy et al. have demonstrated the possibility of using submicron vaterite particles (~500 μm) loaded with porphyrazine anticancer drug as an in vivo theranostic system (Figure 7B) [98]. A high sensitivity of the porphyrazine release to an even slightly acidic pH (6.8) represented a rationale behind the choice of these carriers in their study. Namely, the release of slightly more than 50% of the loaded drug within 3 h was shown there due to the partial carrier dissolution at pH 6.8. Injection of the carriers into the tail vein of tumor-bearing mice resulted in their passive accumulation in the tumor followed by an hours-scale release of the drug, which permeated then to the entire interstitium of the solid tumor. That enabled the intravital imaging and PDT of xenograft tumors.

**Figure 8:**
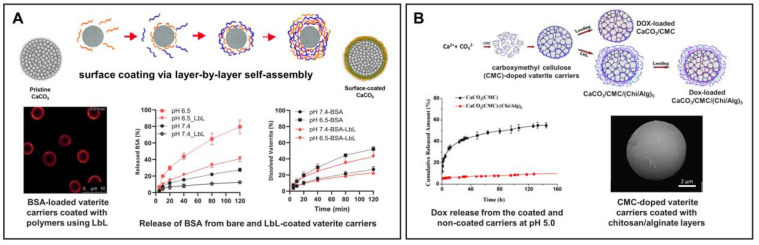
Prevention of the burst release from vaterite carriers via their surface modification. (**A**) Effect of the layer-by-layer (LbL) coating formation on the BSA release from the vaterite carriers: schematics of the polyelectrolyte layers deposition, CLSM image of the LbL-coated carriers and BSA release kinetics from the bare and coated carriers at different pH (6.5 and 7.4). Adapted with permission from [44,127,140]. (**B**) Effect of carboxymethyl cellulose (CMC) incorporation and further coating of the vaterite matrices with chitosan/alginate (Chi/Alg) multilayers on the payload release: schematics, SEM-image and kinetics of DOX liberation from the CMC-doped carriers, both coated and non-coated with Chi/Alg, at pH 5.0. Adapted with permission from [78].

We should note that in the above-mentioned work, the rapid drug release from the vaterite particles was required to provide the high drug concentration in the vessel for creating a gradient from the intracapillary space to the interstitium. However, the need for a more precise control over the payload release encourages researchers to optimize the structure of vaterite-based carriers, including by modifying their surface, for providing better tumor selectivity and prevention of drug liberation in the bloodstream. For instance, Choukrani et al. have synthetized the vaterite nanoparticles loaded with bovine serum albumin (**BSA**) and demonstrated that modification of their surface with carboxyl group-containing polymers using a layer-by-layer (**LbL**) assembly technique could provide their stabilization in neutral aqueous solutions (Tris pH 7.5) postponing the recrystallization from 5 h to 2 months [140]. The investigation of the BSA release kinetics in conditions mimicking the blood flow (flow rate of 0.2 mL min^−1^) demonstrated almost twice reduction of the BSA release from the polymer-coated carriers compared to the pristine particles at pH 6.5 and 7.4 (Figure 8A). The authors suggested that it would ensure the prevention of a burst payload release in the bloodstream; meanwhile, the entry of such carriers into the tumor could trigger drug liberation.

Peng et al. have demonstrated the effect of carboxymethyl cellulose (**CMC**) incorporation into the vaterite matrix on the release of the encapsulated DOX (Figure 8B) [78]. Negatively charged CMC possesses hydrophobic properties at acidic conditions as a result of protonation of the carboxyl groups with subsequent inhibition of the vaterite particles dissolution leading to the slowing down of the DOX release rate at pH 5 (0.1 M sodium citrate–HCl buffer). Further modification of the CMC-containing carriers with chitosan and alginate multilayers via the LbL self-assembly technique allowed the authors to drastically decrease the rate of DOX liberation, when 10% of the loaded drug released at pH 5 during 150 h.

**Figure 9:**
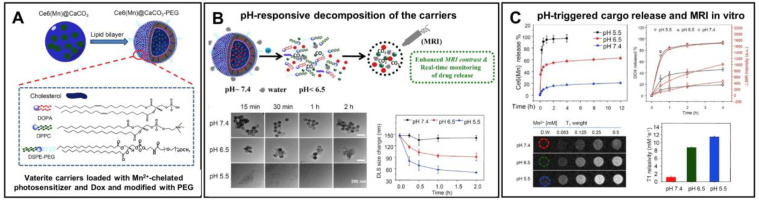
Enhancement of the vaterite carriers targeting to tumors via their surface modification. (**A**) Schematics representing the structure of vaterite carriers loaded with Mn^2+^-chelated chlorin e6 photosensitizer (Ce6(Mn)) and modified with PEG. (**B**) Schematic illustration of the pH-responsive decomposition of the carriers (incubation in PBS at pH 5.5, 6.5 and 7.4). (**C**) pH-triggered MR enhancement and MR-imaging monitored photosensitizer release in vitro. Adapted with permission from [141].

In addition to control over the payload release, surface modification of the vaterite-based carriers with various polymers, antibodies, peptides and aptamers can simultaneously facilitate the drug targeting [18]. Thus, Dong Z. et al. have designed pH-responsive calcium carbonate carriers loaded with a Mn^2+^-chelated chlorin e6 photosensitizer and DOX drug, the surface of which was functionalized with polyethylene glycol (**PEG**) [141]. The carriers demonstrated relatively good stability under physiological pH 7.4 (less than 20% of the loaded drug amount was liberated during 12 h for both therapeutic compounds), but high sensitivity to pH as they were displaying rapid degradation and payload release at acidic conditions (Figure 9). PEGelation provided a sufficient blood circulation time for the carriers injected in tumor-bearing mice in vivo (the first and the second phases of the circulation half-lives were ~1 h and ~14 h, respectively). The designed carriers exhibited a pH-dependent enhancement of the T1-weighted magnetic resonance (**MR**) contrast due to Mn^2+^-chelated photosensitizer liberation at an acidic pH both in vitro and in vivo. This feature allowed the authors to study the efficacy of tumor-targeted delivery for the loaded drugs by means of intravenously injected carriers utilizing MR and fluorescence imaging modalities. As a result, the gradual accumulation of the carriers in the tumor was shown which enabled the effective realization of combined PDT and chemotherapy, which granted the synergistic anti-tumor effect.

**Figure 10:**
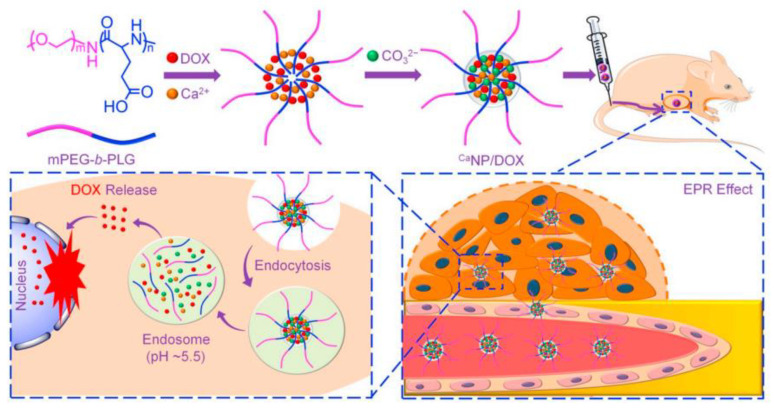
Schematic illustration for fabrication of the DOX-loaded calcium carbonate-crosslinked polypeptide carriers (CaNP/DOX), their circulation in vivo, intratumoral accumulation and pH-triggered intracellular DOX release. Reproduced from Open Access Article [142].

CaCO_3_ particles are effectively integrated with the other encapsulation systems to generate the advanced pH-responsive vaterite-derived platforms. Some interesting examples have been recently reviewed by Tan and co-authors [44]. This review highlighted different polymer-doped vaterite containers, as well as introduced various hybrid systems obtained when integrating CaCO_3_ with emulsions, hydrogels and liposomes. Besides, CaCO_3_ mineralization of the micellar core allows the formation of pH-responsive vehicles, which were demonstrated to be especially valuable in terms of the intracellular delivery of anticancer drugs [142], including the co-delivery of various therapeutic agents [143,144]. Concerning the acidic pH of cellular endosomes (pH 5.5–6.5) and lysosomes (pH 4.5–5.5), such mineralized polypeptide nanoparticles enable the pH-triggered intracellular release of the payload, while protecting it from the leakage at the physiological pH, which extend the circulation half-life and, thus, enhance the drug accumulation in tumors (Figure 10). We will discuss further the other possibilities of controlling the process of decomposition for vaterite-based carriers.

The pH sensitivity of vaterite is also successfully utilized for the development of different antibacterial coatings. This possibility arises due to local acidification (pH 5.0–5.5) of the environment by bacteria during their growth and metabolic processes [145]. Antibacterial film, which was based on vaterite microspheres loaded with a sanguinarine (**SAN**) drug, has demonstrated a strong bactericidal activity against *Staphylococcus aureus* [146]. Lowering the pH from 7.0 to 5.0 upon the film incubation in PBS resulted in the liberation of 46% instead of 21% of the loaded SAN during 67 h. Importantly, when growing the bacteria on the surface of this film, its gradual decomposition was observed. Namely, the coating became transparent with the growth of bacteria as the result of vaterite dissolution. At the same time, a large zone of inhibition was formed indicating the release of SAN from the carriers. The authors suggested that these processes were induced by the acidic environment of bacteria.

**Figure 11:**
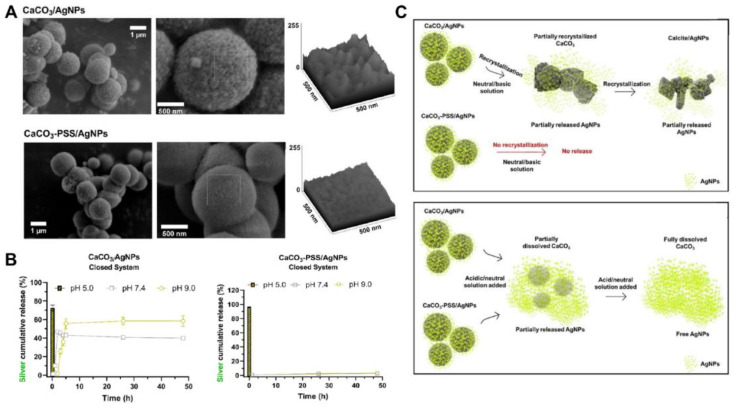
Vaterite–nanosilver hybrids with antibacterial properties and pH-triggered release. (**A**) SEM images and surface roughness plots of the pristine (CaCO_3_/AgNPs) and PSS-modified (CaCO_3_-PSS/AgNPs) vaterite–nanosilver hybrids. (**B**) Cumulative release of silver ions from the hybrids in PBS at pH 5.0, 7.4 and 9.0. (**C**) Schematic illustration of the AgNPs release driven by the recrystallization and dissolution of the hybrids. Adapted from Open Access Article [147].

Ferreira A. et al. have designed pH-sensitive vaterite–nanosilver hybrids, which demonstrated good activity against *Escherichia coli*, methicillin-resistant *Staphylococcus aureus* and *Pseudomonas aeruginosa* [147]. However, the pristine silver-loaded particles were characterized by initial burst release even in non-acidic buffers (at pH 7.4 and pH 9.0) liberating ~50% of the incorporated AgNPs within a few hours (Figure 11). The incorporation of poly(4-styrenesulfonic acid) sodium salt (**PSS**) during the formation of the hybrids allowed the prevention of premature AgNPs release at non-acidic pH. Namely, the PSS-containing carriers did not recrystallize during 50 h at pH 7.4 and pH 9.0, so no AgNP release was observed during this period. In contrast, at pH 5.0 an immediate burst release occurred resulting in the liberation of over 90% of loaded AgNPs from the hybrids.

**Figure 12:**
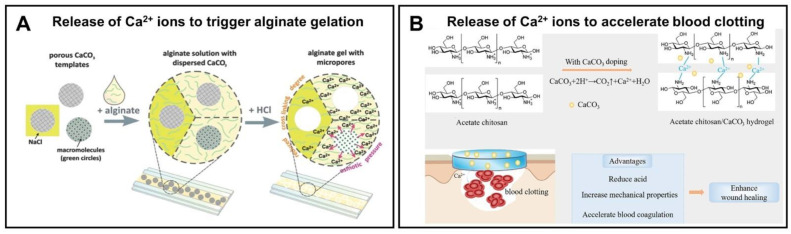
Exploiting of Ca^2+^ release, which occurs during the dissolution of vaterite particles, for triggering the alginate gelation (**A**) and accelerating the blood clotting (**B**). (**A**) reproduced with permission from [55], (**B**) from [149].

Similar to the vaterite–calcite recrystallization process, the decomposition of vaterite carriers at an acidic pH not only induces the payload liberation, but also ensures the release of Ca^2+^ ions. This feature, for example, is often applied to trigger alginate gelation [55,123,148] (Figure 12A). The released Ca^2+^ ions could also participate in hemostasis, catalyzing different coagulation-related reactions that promote the blood coagulation process [18,149,150] (Figure 12B).

**Figure 13:**
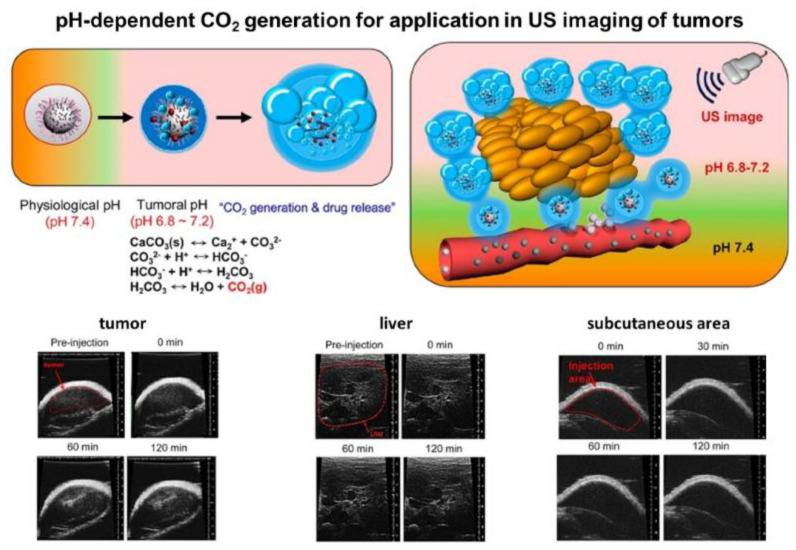
Exploiting of CO_2_ bubbles’ generation, which occurs during the dissolution of vaterite particles, for US imaging of tumors. Schematic illustration of the pH-dependent CO_2_ generation and the images showing the US contrast in tumor, liver and subcutaneous area after the injection of DOX-loaded vaterite carriers in vivo to the corresponding site. Reproduced with permission from [152].

Moreover, during the decomposition in acidic media, vaterite generates carbon dioxide (CO_2_) bubbles that open up the potential of its application in ultrasound (**US**) imaging [151,152], as well as in US cavitation and sonodynamic therapy [45,153]. For instance, Min K.H. with co-authors have shown the possibility to exploit such gas generation in US imaging of tumors [152]. Their DOX-loaded vaterite-based carriers exhibited strong echogenic signals at a tumoral acid pH in vivo in mice through the production of CO_2_ bubbles (Figure 13). Importantly, in normal (non-tumoral) tissues the carriers did not provide any US contrast as no bubble generation occurred there. Furthermore, the DOX release, which was induced by the carriers’ dissolution in the tumor, granted the antitumor therapeutic effect in that study. The proposed concept is very promising as it opens new perspectives for the development of novel theranostic platforms combining ultrasound imaging and therapy for various cancers. Thus, for instance, in further elaboration of this idea, the authors have designed the photosensitizer-loaded vaterite carriers with a potential for US imaging-guided photodynamic destruction of cancer cells [151].

In the work [45], photosensitizer-loaded vaterite carriers were tested for their ability to destruct tumors under the US treatment (0.89 MHz) followed by the light irradiation US of certain intensity producing the acoustic cavitation, which effects, such as the formation of microjets and shock waves [154], can cause cytotoxic effects in tumor cells [155]. Varying the US power density (0.05–1.00 W/cm^2^) and the pH of the immersion medium (7.0 and 5.0), the controlled cavitation-mediated release of aluminum phthalocyanine from the carriers was shown. At pH 7.0, the bubbles’ formation was weakly intense until the power density of sonication reached 1 W/cm^2^. Then, intensification of the bubbling process occurred, also accelerating the vaterite–calcite recrystallization and subsequent liberation of the photosensitizer. At the same time, at pH 5.0, the carrier dissolution accompanied by the payload release and CO_2_ bubbles generation was observed even without the US treatment, while the sonication with the power densities above 0.2 W/cm^2^ drastically intensified these processes. Given the acidity of the tumor microenvironment, the carriers will be dissolved upon the accumulation inside and thus produce CO_2_ bubbles, which generation could be enhanced by the US exposure. In vivo investigation in tumor-bearing rats approved this suggestion, revealing the damaging effect of sonication after the intratumoral injection of the carriers. Further irradiation with a light at the wavelength corresponding to the photosensitizer absorption maximum allowed the enhancement of the therapeutic effect.

**Figure 14:**
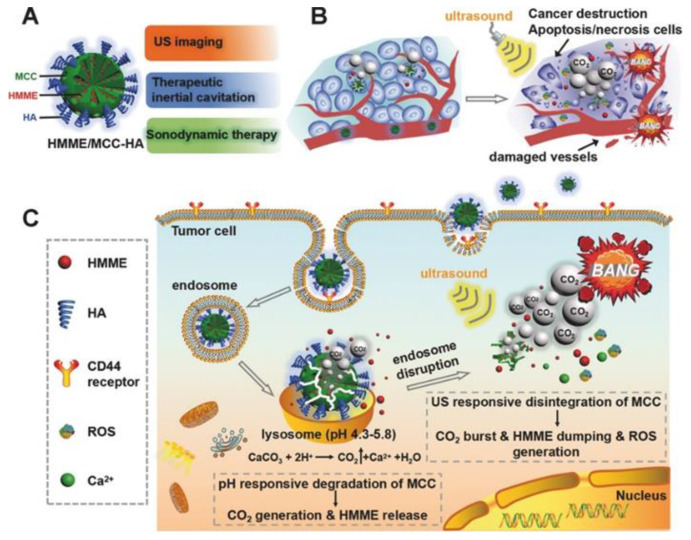
Schematic of the pH/ultrasound dual-responsive CO_2_ generation for US imaging-guided therapeutic inertial cavitation and sonodynamic therapy. (**A**) Formation of the hematoporphyrin monomethyl ether (HMME)-loaded vaterite carriers coated with hyaluronic acid (HA). (**B**,**C**) Mechanism of the tumor destruction utilizing the carriers and US treatment. Reproduced with permission from [153].

Following this approach, Feng Q. et al. introduced the vaterite-based carriers capable of decomposing in a tumor under the combined action of an acidic pH and US irradiation as a result of the simultaneous release of the loaded drug and CO_2_ bubbles’ generation (Figure 14) [153]. That led to cavitation-mediated irreversible necrosis of tumor cells and destruction of its blood vessels. To achieve the anticancer synergism, the carriers were loaded with a sonosensitizer; thus, they could provide the reactive oxygen species generation leading to apoptotic destruction of the cancer cells. Moreover, the echogenic property of CO_2_ provides the US imaging guidance for therapeutic inertial cavitation and sonodynamic therapy simultaneously.

To date, a great number of different pH-responsive CaCO_3_-based delivery systems and composites have been designed for anticancer, antibacterial and other drug encapsulation. Recent advances in this field have been discussed in a number of well-organized and comprehensive reviews [3,18,58,59,61,156]. Such microenvironment-activated systems are mainly applied in chemotherapy, photothermal therapy or PDT, wound healing, blood clotting, tissue engineering, as well as in ultrasound, fluorescence and MRI imaging [18]. Both relatively simple vaterite-based systems and composite multicomponent platforms, which are highly demanded in multimodal theranostics, find their application in biomedicine.

## 5. Biodegradation of Vaterite Carriers

Vaterite-based drug carriers exhibit high biocompatibility and good biodegradability participating in the normal metabolism of the living body by dissolving into nontoxic ions. There are two possible routes for the calcium-based materials degradation: the dissolution by body fluids, and phagocytosis and absorption by cells (mainly macrophage) [157]. The first route includes a split of the carbonate materials into particles, molecules or ions due to the acidic environment of the body fluids containing a number of acidic metabolites such as citrate, lactate and acid hydrolysis enzyme. The second route can be divided into intracellular and extracellular degradation, where the particles can be split into ions after phagocytosis by macrophages under the effect of cytoplasmic and lysosomal enzymes, and then the degradation products, such as Ca^2+^ and CO_3_^2−^, can be transferred to outside the cell. Additionally, the environment of macrophages enriched with acid hydrolases (including lysosomal enzyme and acid phosphatase enzymes) promotes a secretion of H^+^ and induces the pH decrease.

Fu K. et al. demonstrated the biodegradation of the composite comprising a calcium carbonate scaffold enveloped by a thin layer of hydroxyapatite [158]. Despite the slow biodegradability of hydroxyapatite, the complete resorption and remodeling of the implanted calcium carbonate-based composite takes 18–24 months, which was revealed by in vivo clinical observations. Moreover, the promotion of conductive osteogenesis was assessed in vitro by the successful attachment and proliferation of human mesenchymal stem cells on the composite and in vivo using an immunodeficient mouse model.

**Figure 15:**
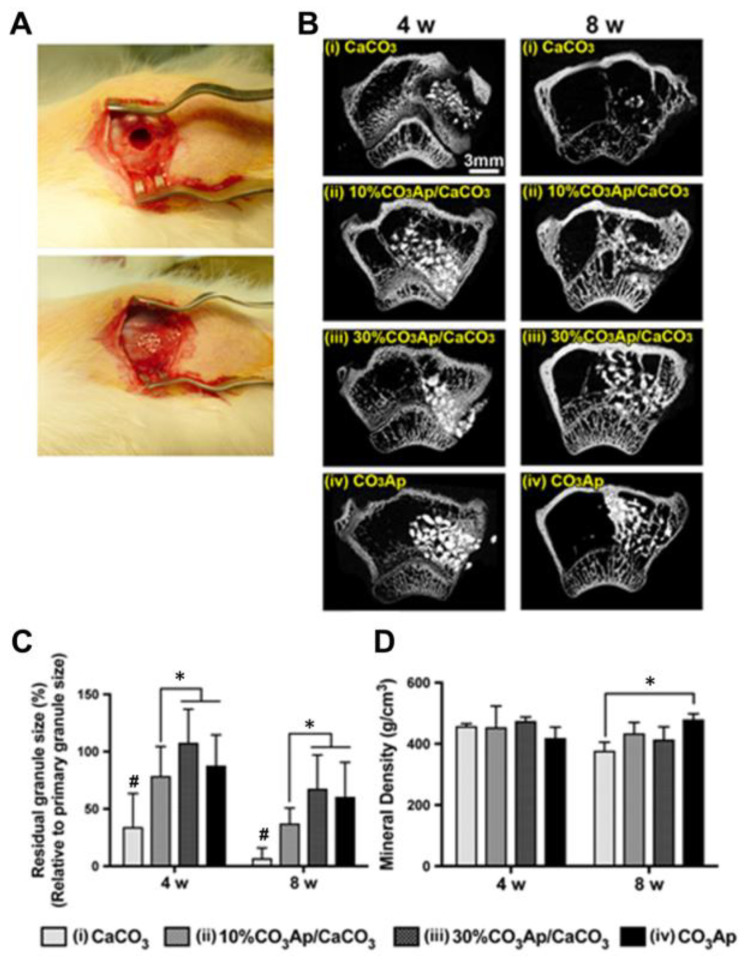
In vivo degradation of the CaCO_3_-based implanting material for improvement of ossification in bone tissue engineering. (**A**) Implantation of the fabricated granules in cylindrical bone defects of the rabbit femur. (**B**) Horizontal μCT views of the rabbit femur defect with CaCO_3_, 10% CO_3_Ap/CaCO_3_, 30% CO_3_Ap/CaCO_3_ and CO_3_Ap granules at 4 and 8 weeks. (**C**) The residual granules area (%) quantified by μCT at 4 and 8 weeks. (**D**) Mineral density the bone defect area at 4 and 8 weeks. An asterisk (*) denotes significant differences between groups, *p* < 0.05; a hash (#) denotes significantly lower than all other modalities, *p* < 0.05. Reproduced with permission from [161].

The metabolites of vaterite degradation can participate in the formation of new bone, thus completing the transformation of inorganic materials in organisms. Stengelin E. et al. successfully applied the conversion of vaterite to bone-like hydroxycarbonate apatite (**HCA**) under physiological conditions in the development of bone scaffolds based on biodegradable vaterite/PEG-composite microgels [159]. FT-IR spectroscopy indicated the transformation of vaterite in the polymer matrix to HCA, and co-encapsulation of vaterite with the osteoblast cells (MG-63 GFP) characterized by a similar cell viability and high cell compatibility compared to a microgel containing only cells without vaterite. The application of calcium carbonate implants in rabbit bone defects revealed their rapid degradation even before osteoconduction was completed [160]. The results indicated abundant woven bone in the cortical shell of the surgical site, indicative of spontaneous healing without an osteoconductive implant. To prolong the osteoconductivity, Fujioka-Kobayashi M. et al. used CaCO_3_ core coated with carbonate apatite [161]. The biodegradation of CaCO_3_ is caused by dissolution or cell mediation depending on the mineral phase [162], while calcium phosphate biomaterials resorption is associated with the combination of physical, chemical and biological processes [163]. The combination of CaCO_3_ and carbonate apatite allowed the authors to balance new bone formation and material resorption leading to suitable bone replacement, where the higher contents of CaCO_3_ resulted in a shortened resorption rate with the subsequent promotion of Ca^2+^ release and carbonate apatite and, in turn, demonstrated a perfect osteoconductive potential (Figure 15). Another work described the hybrid system composed of the vaterite particles formed in the presence of inorganic polyphosphate (**polyP**), which restrain vaterite–calcite recrystallization [164]. The hybrid particles degraded within 5 days of the incubation in the cell culture medium with 65% of suppression of calcite formation in the first 3 days. The rapid degradation of CaCO_3_/polyP particles was confirmed by a Ca^2+^ release investigation portraying 68% of the total Ca^2+^ in the reaction mixture compared to almost no Ca^2+^ content for the calcite sample.

Unger R. et al. [165] declared the in vivo biodegradation of an injectable bone substitute composed of PEG-acetal-dimethacrylate and vaterite nanoparticles mediated by mononuclear cells of the macrophage lineage via a pro-inflammatory process. During degradation of the material, M1 macrophages involved in this process may express lytic enzymes such as the members of the group of reactive oxygen species and other relevant mediators [166].

The mechanism of the in vitro resorption of natural CaCO_3_ by avian osteoclasts was investigated by Guillemin et al. demonstrating that carbonic anhydrases produced by osteoclasts play a crucial role in generating protons for the acidification of the calcium carbonate [167]. The calcium carbonate from *Tridacna* shell is a biomaterial that can undergo dissolution through the mechanism of osteoclastic resorption. The degradation of the carbonate-based materials induced by bacterial activity was demonstrated in [168]. In aerobic systems, the decomposition of CaCO_3_ is attributed to a metabolic byproduct through the bacterial-induced decomposition of skeletal-binding organic matter.

**Figure 16:**
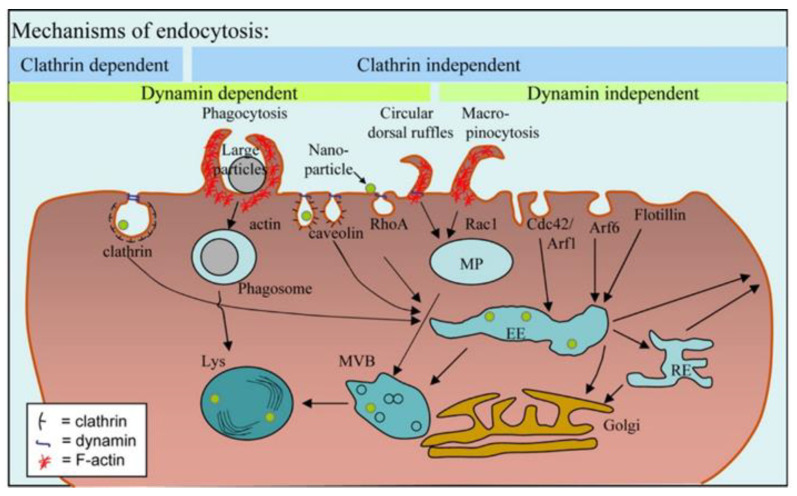
The scheme of the micro- and nanoparticle internalization by endocytic mechanism and intracellular transport. Reproduced with permission from [169].

The pH-dependent biodegradation of vaterite nanoparticles was discussed in the case of the drug delivery to tumors, where the authors concluded that the blood flow rate plays a crucial role in this process [98]. Different perfusion rates influence the pH of tumor venous blood from neutral to acidic values resulting in the partial or complete degradation of the vaterite. Moreover, it is shown that the main pathway of the CaCO_3_ particles internalization is micropinocytosis with their subsequent resorption in lysosomes, which are characterized by an acidic pH (4.0–5.5) [169] and good stability in the extracellular space for longer times (Figure 16).

**Figure 17:**
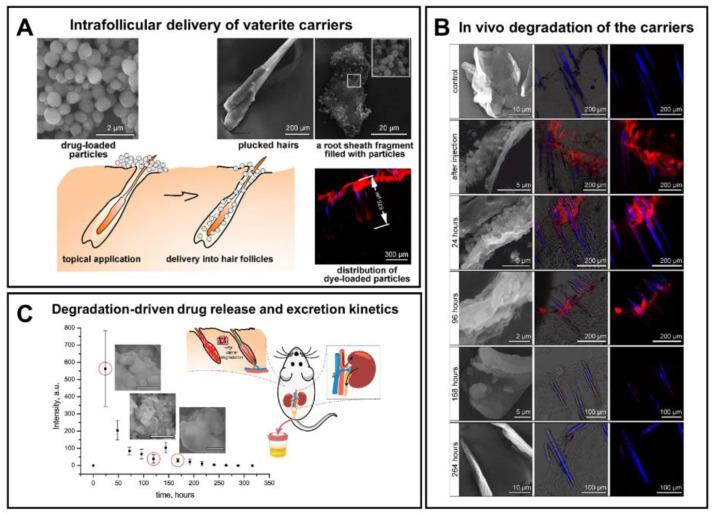
In vivo degradation of vaterite carriers in hair follicles. (**A**) SEM, CLSM images and schematics illustrating the intrafollicular delivery of the carriers. Reproduced with permission from [170]. (**B**) SEM (**the left column**) and CLSM (**the middle and right columns**) images illustrating the process of the carriers’ degradation inside the hair follicles of rats in vivo. (**C**) Excretion kinetics of the fluorescent dye intrafollicularly delivered by means of degradable vaterite carries. (**B**) and (**C**) reproduced with permission from [16].

In vivo degradation of the vaterite carriers was demonstrated in the rat skin, both after their delivery to the dermis using fractional laser microablation (**FLMA**) [15] and after non-invasive intrafollicular administration of the carriers [16]. In the first case, the carrier degradation was enhanced by the inflammatory reaction occurred in skin as a result of the FLMA-microchannels’ formation. Meanwhile, in the second case, the hair cycle stimulated processes, which activated the secretion within the hair follicle forming the release medium for the particles and delivering drugs. That led to a gradual degradation of the vaterite particles inside the hair follicles, which ended up with their total resorption within 12 days (Figure 17). The biodegradation of the vaterite particles was followed by the in situ release of the payload ensuring its distribution in the hair follicle tissue and subsequent systemic uptake.

**Figure 18:**
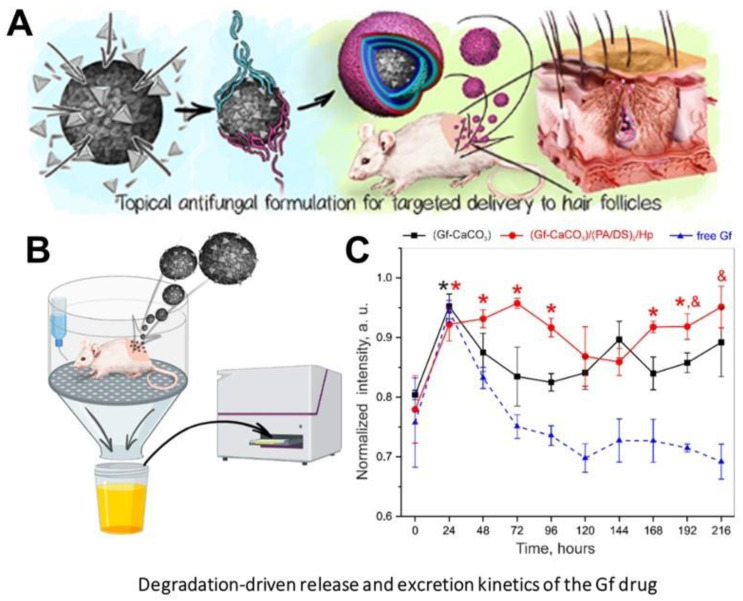
Prolongation of in vivo degradation of the vaterite carriers and sustainment of the payload release via formation of the stabilizing coating on the carriers’ surface. (**A**) Schematics illustrating the formation of vaterite carriers loaded with a griseofulvin (Gf) drug and coated with poly-L-arginine (PA), dextran sulfate (DS) and heparin (HP) polyelectrolytes. (**B**) Schematics of the Gf urinary excretion rate investigation. (**C**) Urinary excretion profiles of Gf after its administration by means of (Gf-CaCO_3_) and (Gf-CaCO_3_)/(PA/DS)_2_/HP carriers or after pure Gf application in rats in vivo. An asterisk (*) indicates significant differences in the Gf peak intensity at a particular time point after drug delivery as compared to the control urine value (zero time point) within the same group (*p* < 0.05). An ampersand (&) shows a significant difference in the Gf peak intensities between the group of (Gf-CaCO_3_)/(PA/DS)_2_/HP carriers and the pure Gf group (*p* < 0.05) on the last day of the experiment. Reproduced with permission from [43].

As a practical implementation of intrafollicular drug transportation following the vaterite degradation, such carriers were applied for influenza vaccine delivery proposing the new strategy for transcutaneous immunization [90], as well as for the photosensitizer targeting enabling the improvement of psoralen–ultraviolet A therapy of dermatoses [171,172]. Besides, the vaterite particles were applied for the intrafollicular delivery of antifungal drugs [43,101,113,173]. In particular, the immobilization of a griseofulvin drug (**Gf**) into such biodegradable carriers enabled its dermal bioavailability enhancement [43,113]. The degradation-driven liberation of the loaded Gf from the carriers was evaluated in water, saline and cell culture medium [43]. The influence of the release medium has a dramatic effect on the degradation rate of the vaterite matrix driven mainly by its transition to calcite. The acceleration of the CaCO_3_ recrystallization process was demonstrated in saline caused by its higher ionic strength, speeding up ion exchange between the CaCO_3_ surface and the incubation solution. Oppositely, the incomplete degradation of carbonate carrier in cell culture medium was attributed to the adsorption of protein molecules from this medium on the carrier surface. The modification of the particle surface with polyelectrolyte shell (poly-L-arginine, dextran sulfate and heparin) via the LbL approach extended the recrystallization duration for Gf-loaded carriers twice in water (144 h vs. 72 h) and 2.5 times in saline (120 h vs. 48 h). The sustained effect of the stabilizing shell was also verified in vivo, when delivering these carriers into the hair follicles of rats (Figure 18). According to the drug excretion profiles, the use of such a formulation provided detectable Gf concentrations in urine for over a week (168–192 h). Importantly, no obvious adverse effects were observed upon the multi-dose dermal toxicity assessment of the Gf-loaded vaterite carriers in rabbits, while a high antifungal efficiency was demonstrated when studying their therapeutic potential in a guinea pig model of trichophytosis [113]. This methodology was extended to deliver the antifungal drug naftifine hydrochloride into the deep layers of the skin through the hair follicles [101]. Scanning electron microscopy (SEM) investigation revealed the vaterite bulk resorption within 72 h inside the follicles of mice followed by its gradual degradation within 120 h with the simultaneous release of the payload drug into the surrounding tissues. To accelerate the vaterite carrier degradation in skin, the authors in Ref. [174] proposed an application of sonophoretic post-treatment (1 MHz, 1 W/cm^2^, 9 min) after the particles’ delivery into hair follicles. Theresults of optical coherence tomography monitoring of the skin and SEM investigation of the plucked hairs revealed the twice-reduction of the degradation period of the carriers.

## 6. Control over the Dissolution/Recrystallization/Degradation Process of Vaterite

Various applications of vaterite require its stabilization to prevent degradation/recrystallization in aqueous environments, including implantable drug delivery systems, tissue engineering platforms, food/cosmetic additives and storage materials, which are designed for prolonged action [175]. As mentioned above, the regulation of the CaCO_3_ stability could be driven by the addition of macromolecules of a different nature during the particle synthesis or by the CaCO_3_ surface modification by the polymer film. The polymer network suppresses the ions diffusion from the carbonate crystal surface, which resulted in the stabilization of vaterite nanocrystals.

**Figure 19:**
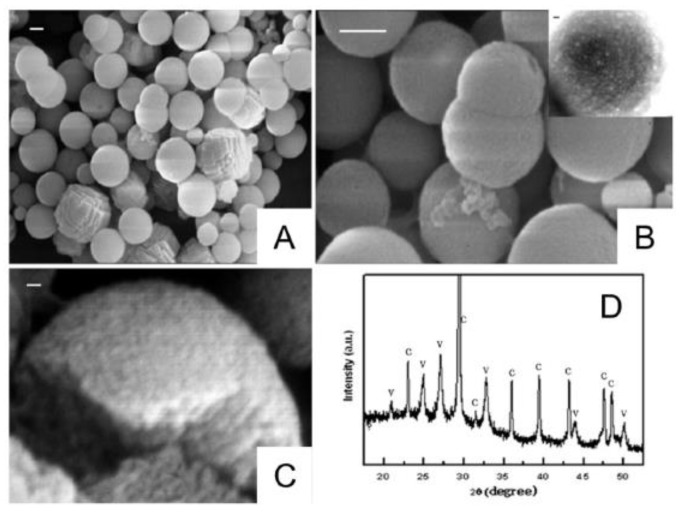
Stabilization of metastable vaterite in CaCO_3_ biomineralization through the addition of ovalbumin protein. (**A**–**C**) Calcium carbonate particles formed in the presence of 0.2 gL^−1^ ovalbumin. (**D**) XRD spectrum of the CaCO_3_-ovalbumin precipitates, where “C” indicates calcite peaks (JCPDS: 05-0586), “V”—vaterite ones (JCPDS: 33-0268). Scale bars correspond to 1 μm (**A**,**B**) and 100 nm (**C**,**D**). Reproduced with permission from [26].

The different additives were applied to control the degradation/recrystallization of the vaterite particles. So, the amino acids and polypeptides were found to have a pronounced effect on the stabilization of the vaterite polymorphs. The presence of polar C=O groups in the structure of amino acids has a crucial influence on the electrostatic interactions of Ca^2+^ ions with the negatively charged oxygen atoms within the C=O bonds, which along with the diffused CO_3_^2−^ ions toward the fixed Ca^2+^ may initiate the critical nuclei of vaterite formation [176]. Thus, the supersaturated solutions of lysine, glycine, alanine, polyglycine, polymethionine, polylysine and polyaspartate were demonstrated to control the vaterite recrystallization [176,177]. In [26], the authors demonstrated the stabilizing effect of negatively charged ovalbumin over positively charged lysozyme to prevent the metastable vaterite from transformation via dissolution-recrystallization processes. The results confirmed that only the net of negatively charged proteins enhance its stability as a result of the strong binding between carboxylate groups of ovalbumin and the calcium ions on the CaCO_3_ surface (Figure 19).

Similar results were shown for the particles co-precipitated with BSA, where the formation of stable vaterite was attributed to the interaction of BSA functional groups (namely, C=O, HO-, N-H, C-N) with the carbonate surface [178]. In another study, the proteins extracted from gastroliths of the crayfish *C. quadricarinatus* induced the stabilization of amorphous calcium carbonate (**ACC**) in vitro mediated by the phosphorylated residues of phosphoproteins [179]. The major proteinaceous fraction of the organic matrix with a heavily phosphorylated doublet band at 70–75 kDa was also incorporated into the mineral phase during the precipitation. The single amino acids, phosphoserine or phosphothreonine, have a similar stabilizing effect proving that phosphoamino acid moieties are the key factors in the control of ACC formation and stabilization.

**Figure 20:**
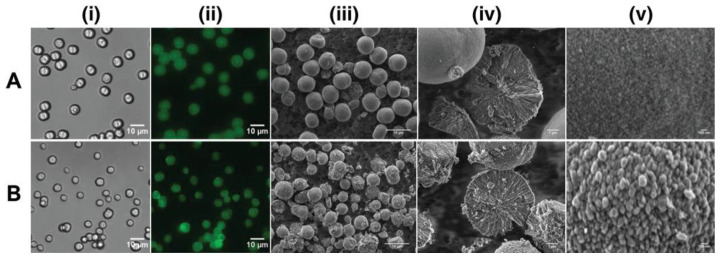
CLSM and SEM images of carboxymethyl–dextran–FITC/vaterite hybrids (**A**) and diethylaminoethyl–dextran–FITC/vaterite hybrids (**B**). Scale bar is 10 µm for (**i**), (**ii**) and (**iii**), 1 µm for (**iv**), and 100 nm for (**v**).Reproduced with permission from [175].

Among polysaccharides, the incorporation of dextran and its derivatives into the vaterite particles by co-precipitation revealed the possibility of their selective stabilization depending on the polymer charge [175]. The co-synthesis of vaterite with the nonionic dextran resulted in the decreasing of the nanocrystallite size with partial blocking of the crystal pores. The inclusion of negatively charged carboxymethyl-dextran significantly retarded the vaterite–calcite recrystallization under a basic pH, whilst positively charged diethylaminoethyl–dextran did not affect this process (Figure 20).

**Figure 21:**
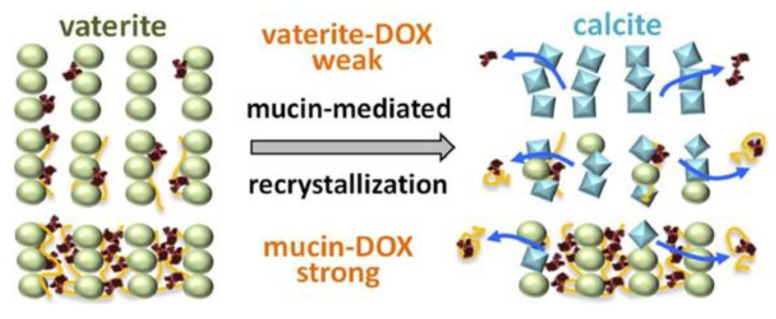
Scheme illustrating the process of vaterite stabilization by mucin incorporation. Reproduced with permission from [83].

Similar observations were demonstrated in the co-synthesis of calcium carbonate with the anionic functional biopolymer carboxymethylinulin, which resulted in enhanced stability of the vaterite phase [180]. Elsewhere, the degradation of vaterite was suppressed by co-precipitation with mucin, as glycoprotein possesses functional groups of different charge [83]. The filling of porous vaterite crystals with a gel-like matrix of mucin reduced ion mobility near the crystal surface in aqueous solution and hampered the recrystallization rate of vaterite to calcite. An increase of mucin content in the obtained hybrid particles reduced the release rate of the encapsulated cationic drug DOX via stabilization of the porous vaterite crystals against recrystallization to non-porous calcite (Figure 21).

The carbonate controlled-addition method was applied to synthesize poly(acrylic acid) (**PAA**)–calcium carbonate composite particles, which were extremely stable in the aqueous medium [181]. The stabilization of ACC was achieved by the complexation of Ca^2+^ ions with PAA and dependent on the polymer molar mass and duration of the complexation process. The shorter complexation time together with the usage of medium molar mass PAA induced the stabilization of ACC due to a more random coordination of Ca^2+^ ions with PAA. In other work, the stability of polycrystalline vaterite was achieved due to the specific interaction between poly(vinyl alcohol) and CaCO_3_ through hydrogen-bonding, probably to the carbonate ions, allowing a high density of polymer chains right near the interface by increasing the number of segments that are intimately attached to the solid [182]. The incorporation of poly (vinylsulfonic acid) through co-synthesis with calcium carbonate controls growth of the vaterite polymorph and its stability over degradation owing to sequestering calcium ions followed by slowing down the nucleation rate and preventing surface calcification or aggregation into microparticles [183]. The obtained vaterite maintained a crystal structure for about 5 months of storage in aqueous medium. An interesting approach was proposed to obtain vaterite particles with long-term stability via the addition of Ca(OH)_2_ to branched polyethylenimine (**PEI**)–CO_2_ adduct solutions [184]. The hydrolysis of the alkylammonium carbamate zwitterions in PEI-CO_2_ adducts led to the release of bicarbonate ions to feed the in situ vaterite crystal nucleation and growth, thus serving as both the CO_2_ source and template for vaterite CaCO_3_ nucleation and growth. The synthesized particles retained the vaterite phase for at least 8 months of storage.

The vaterite crystalline dissolution and recrystallization could be inhibited by the inclusion of the polycarboxylate-type superplasticizer (**PCS**) during its synthesis [185]. The crystal growth process of vaterite microspheres was assisted by the PCS molecules, which rearranged on the surfaces of the vaterite particles and modulated the formation of lenticular aggregates through the hydrogen bonding effect. The carboxylate groups of the polymer interacted with Ca^2+^ ions blocking the transformation of vaterite to calcite. The authors of [186] presented the stabilized vaterite by the poly(amidoamine) dendrimers with external carboxylate groups. The control over dissolution was achieved for more than 1 week at storage in water. The increase of the -COONa groups concentration and the generation number of the dendrimer resulted in the reduction of the vaterite size.

**Figure 22:**
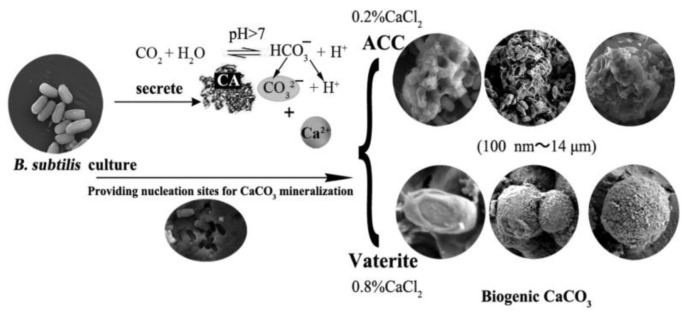
Schematics of possible mechanisms of CaCO_3_ biomineralization. Reproduced with permission from [187].

An interesting research study introduced the application of *B. subtilis* bacterial cells as the templates to the formation of biogenic ACC or vaterite, where the carbonic anhydrase secreted by the bacteria plays an important role in the mineralization of CaCO_3_ [187]. The results indicated the growth of the vaterite phase only in the presence of carbohydrate (crude extracellular protein contains some polysaccharide), whilst both polysaccharides and proteins secreted by bacterial metabolism maintained the stability of vaterite (Figure 22). Other biotic experiments explained the long-term stabilization of vaterite due to binding between the vaterite surface and organic by-products of bacterial activity (extracellular polymeric substances, which include polysaccharides, proteins, glycoproteins, nucleic acids, phospholipids and humic acids) [188].

## 7. Conclusions

The development of degradable systems for biomedical applications eliminates the need to retrieve or dispose of them once their function has been fulfilled. Nowadays, the amount of different materials available for this purpose is immense. Having the lack of the toxicity, expensive production and sophisticated degradability, calcium carbonate in its vaterite polymorph is highly attractive to the design of such systems. The metastability of vaterite manifests in its recrystallization into non-porous calcite crystals via dissolution–reprecipitation or even complete dissolution, depending on the surrounding medium. Because the environment of the living body in different organs and tissues varies in pH, ionic strength, as well as in the presence of molecules, degradation of the vaterite-based carriers is realized differently providing the sustained release of the incorporated cargo driven by such transitions. This manuscript discussed the benefits of vaterite instability including dissolution at an acidic pH, biodegradation at in vivo conditions, transformation to non-porous calcite and the main approaches to control over these processes. The additional profits of CaCO_3_-based systems include the regulation of the payload liberation by means of their surface modification or accelerated by means of external treatment, e.g., ultrasonication, microwave irradiation etc. Vaterites being a versatile platform can be utilized both in its native form and as part of complex hybrid systems. Additional functionalization with different photo- [38,114,151] and sonosensitive drugs [153], as well as with metallic nanoparticles (silver, gold, magnetite, etc.) [9,69,189,190,191,192,193], enables the creation of multimodal theranostic platforms capable of different biomedical applications. The degradation of CaCO_3_ at mild conditions determines its widespread use for the formation of the polyelectrolyte hollow capsules and core/shell containers by layer-by-layer assembly technique. Besides the transition-driven drug release property, the vaterite particles can serve as a source of Ca^2+^ ions, which are found to be effective in the scaffolds for bone and tooth tissue regeneration due to the ability of calcium ions to improve osteo- and odontoblasts’ activity. An interesting research trend is associated with creation of vaterite-based active colloids (also called micro/nano-motors or swimmers), which exhibit propulsion by transforming energy from their environment into enhanced diffusive motion [194,195,196,197]. The formation of any anisotropy on the particles’ surface (e.g., by its partial coverage with silica layer) enables the generation of self-propulsion upon the vaterite decomposition in acidic media [196]. The ability of vaterite to locally increase the pH medium during its dissolution deserves particular attention. This feature provides the in vivo pH modulation of solid tumors with the selective localization of vaterite particles with distinct sizes (20, 100 and 300 nm) in the extracellular region of tumors, followed by buffering of their environment which resulted in the prevention or reduction of tumor growth [56]. The size-dependence behavior of alkalinization of the acidic pH of human fibrosarcoma (HT1080) cells demonstrated the most pronounced ΔpH and longest effect for the 100 nm particles, while larger (300 nm) and smaller (20 nm) ones were less efficient due to limited diffusion and transient retention in the tumor environment, respectively. The effectiveness of nano-CaCO_3_ was confirmed against RFP-expressing breast cancer cells (MDA-MB-231) without an impact on the growth and behavior of the surrounding fibroblasts [57]. Co-incubation of MDA-MB-231 with fibroblasts with subsequent vaterite treatment indicated the selective inhibition of the MDA-MB-231 cells growth with severe suppression of their cellular migration (which increases by co-incubation with fibroblasts) without affecting the stromal cells. The authors highlighted that this approach could serve as a treatment paradigm for long-term tumor static therapy. Based on the discussed facts, vaterites are not only perfect carriers for various bioactive molecules, but also are of paramount importance in their initial state for the biomedical applications.

The discussed benefits of vaterite’s instability justify its favorable use in the design of multipurpose degradable systems for biomedical purposes. Taking into account the diversity of techniques enabling the synthesis of vaterite carriers in scalable [198] and even automated [199,200] ways, one can consider these systems as especially beneficial for potentially resolving a key bottleneck in industrial applications.

## Data Availability

The data presented in this study are available in this paper.

## Abbreviations

**ACC**	amorphous calcium carbonate
**BSA**	bovine serum albumin
**CD**	cyclodextrin
**CLSM**	confocal laser scanning microscopy
**CMC**	carboxymethyl cellulose
**DOX**	doxorubicin
**EDX**	energy-dispersive X-ray spectroscopy
**Gf**	griseofulvin
**LbL**	layer-by-layer
**MR**	magnetic resonance
**PCL**	polycaprolactone
**PDT**	photodynamic therapy
**PEG**	polyethylene glycol
**SEM**	scanning electron microscopy
**US**	ultrasound
